# Forty years of the adrenal chromaffin cell through ISCCB meetings around the world

**DOI:** 10.1007/s00424-023-02793-0

**Published:** 2023-03-08

**Authors:** Victoria Maneu, Ricardo Borges, Luis Gandía, Antonio G. García

**Affiliations:** 1 Departamento de Óptica, Farmacología y Anatomía, Universidad de Alicante, Alicante, Spain; 2 Unidad de Farmacología, Departamento de Medicina Física y Farmacología, Facultad de Medicina, Universidad de La Laguna, Tenerife, Spain; 3 Instituto Fundación Teófilo Hernando, Madrid, Spain; 4 Departamento de Farmacología y Terapéutica, Universidad Autónoma de Madrid, Madrid, Spain; 5 Facultad de Medicina, Instituto de Investigación Sanitaria del Hospital Universitario La Princesa, Universidad Autónoma de Madrid, Madrid, Spain

**Keywords:** Chromaffin cell, Adrenal medulla, SNARE proteins, Fusion pore, Calcium dynamics, Membrane fusion, ISCCB meetings

## Abstract

This historical review focuses on the evolution of the knowledge accumulated during the last two centuries on the biology of the adrenal medulla gland and its chromaffin cells (CCs). The review emerged in the context of a series of meetings that started on the Spanish island of Ibiza in 1982 with the name of the International Symposium on Chromaffin Cell Biology (ISCCB). Hence, the review is divided into two periods namely, before 1982 and from this year to 2022, when the 21st ISCCB meeting was just held in Hamburg, Germany. The first historical period extends back to 1852 when Albert Kölliker first described the fine structure and function of the adrenal medulla. Subsequently, the adrenal staining with chromate salts identified the CCs; this was followed by the establishment of the embryological origin of the adrenal medulla, and the identification of adrenaline-storing vesicles. By the end of the nineteenth century, the basic morphology, histochemistry, and embryology of the adrenal gland were known. The twentieth century began with breakthrough findings namely, the experiment of Elliott suggesting that adrenaline was the sympathetic neurotransmitter, the isolation of pure adrenaline, and the deciphering of its molecular structure and chemical synthesis in the laboratory. In the 1950s, Blaschko isolated the catecholamine-storing vesicles from adrenal medullary extracts. This switched the interest in CCs as models of sympathetic neurons with an explosion of studies concerning their functions, i.e., uptake of catecholamines by chromaffin vesicles through a specific coupled transport system; the identification of several vesicle components in addition to catecholamines including chromogranins, ATP, opioids, and other neuropeptides; the calcium-dependence of the release of catecholamines; the underlying mechanism of exocytosis of this release, as indicated by the co-release of proteins; the cross-talk between the adrenal cortex and the medulla; and the emission of neurite-like processes by CCs in culture, among other numerous findings. The 1980s began with the introduction of new high-resolution techniques such as patch-clamp, calcium probes, marine toxins-targeting ion channels and receptors, confocal microscopy, or amperometry. In this frame of technological advances at the Ibiza ISCCB meeting in 1982, 11 senior researchers in the field predicted a notable increase in our knowledge in the field of CCs and the adrenal medulla; this cumulative knowledge that occurred in the last 40 years of history of the CC is succinctly described in the second part of this historical review. It deals with cell excitability, ion channel currents, the exocytotic fusion pore, the handling of calcium ions by CCs, the kinetics of exocytosis and endocytosis, the exocytotic machinery, and the life cycle of secretory vesicles. These concepts together with studies on the dynamics of membrane fusion with super-resolution imaging techniques at the single-protein level were extensively reviewed by top scientists in the field at the 21st ISCCB meeting in Hamburg in the summer of 2022; this frontier topic is also briefly reviewed here. Many of the concepts arising from those studies contributed to our present understanding of synaptic transmission. This has been studied in physiological or pathophysiological conditions, in CCs from animal disease models. In conclusion, the lessons we have learned from CC biology as a peripheral model for brain and brain disease pertain more than ever to cutting-edge research in neurobiology. In the 22nd ISCCB meeting in Israel in 2024 that Uri Asheri is organizing, we will have the opportunity of seeing the progress of the questions posed in Ibiza, and on other questions that undoubtedly will arise.

## Introduction

In mammals, the adrenal gland is a double-endocrine organ located on top of both kidneys. The outer part of the gland is the cortex, where corticoid hormones are synthesized and released; the adrenal medulla is the inner part, a tissue formed by chromaffin cells (CCs) that synthesize, store, and release the catecholamines adrenaline and noradrenaline. Both cortex and medulla contribute critically to the body’s response to stress.

The adrenal medulla has been thoroughly studied from a physiological perspective, as it behaves as an amplifying arm of the hypothalamus-sympathoadrenal axis that regulates a myriad of cardiovascular and metabolic processes. Of particular relevance is the explosive release of adrenaline from adrenal CCs, occurring during the fight-or-flight response during acute stress, described by Walter Cannon in 1929 [26].

Having its origin in the neural crest, the adrenal medulla and its CCs have been extensively used as neuronal models to study cell excitability, ion channels calcium signaling, exocytosis, endocytosis, membrane fusion, and the formation and expansion of the fusion-pore. Many concepts on the functioning of synapses in the central nervous system were initially conceived based on experiments carried out in CCs, in laboratories from all over the world.

In the early1980s, new electrophysiological and imaging techniques were introduced. At the same time, the primary cultures of adrenal CCs were initially developed by Bruce Livett [114]. These coincident advances potentiated the interest in using CC cultures to study many basic aspects of exocytotic neurotransmitter release. In this context, a group of scientists interested in this field decided to create a monographic biennial meeting, the so-called International Symposium of Chromaffin Cell Biology (ISCCB). The first meeting was held in Ibiza (Spain) in 1982. The 21st ISCCB meeting has just been held in Hamburg (Germany), in the summer of 2022. In the frame of this meeting, many of the attendants did not know the origin of ISCCB meetings as the founders were retired. Thus, in Hamburg, one of the authors of this review (AGG) was asked to review the history of these meetings to secure their continuity and to follow the further evolution of the field.

So, this review concerns the historical evolution of concepts and ideas around the adrenal medulla and its CCs, in the context of the 21st ISCCB meetings during the last 40 years (Table 1). Additionally, to know the state-of-the-art on the biology of CCs when the first meeting took place in 1982, we considered it useful to include a brief account of the knowledge accumulated from 1852 to 1982, when the Ibiza meeting was held. Then, we will succinctly review the cumulative knowledge on the biology of CCs along the 40 years of ISCCB meetings, with an emphasis in the frontier knowledge on membrane fusion, the fusion pore, and exocytosis emanating from the 2022 Hamburg ISCCB-21st meeting. Most of this knowledge has been described in detail in a recent comprehensive review [28].

**Table 1:** Time, countries, and organizers of the International Symposia on Chromaffin Cell Biology (ISCCB)

Symposium number	Year	Venue	Organizer
1	1982	Ibiza (Spain)	Antonio G. García Valentín Ceña
2	1984	Colmar (France)	Dominique Aunis
3	1986	Coolfont,WV (USA)	Harvey Pollard Patrick Fleming
4	1987	Alice Springs (Australia)	Bruce G. Livett
5	1989	Jerusalem (Israel)	Kurt Rosenheck
6	1991	Marburg (Germany)	Manfred Gratzl Klaus Unsicker
7	1993	Le Château Montebello, Québec (Canada)	José María Trifaró
8	1995	Edinburg, Scotland (UK)	John H Pillips Daniel K Apps
9	1997	Sapporo (Japan)	Tomio Kanno Yoshikazu Nakazato Konosuke Kumakura
10	1999	Bergen (Norway)	Karen B. Helle Torgeir Flatmark Guldborg Serck-Hanssen
11	2001	San Diego, CA (USA)	Daniel O´Connors Sushil Mahata
12	2003	La Palma, (Spain)	Ricardo Borges
13	2005	Pucón (Chile)	Ana M. Cárdenas
14	2007	Sestri Levante (Italy)	Emilio Carbone
15	2009	Mérida (México)	Arturo Hernández-Cruz
16	2011	Beijing (China)	Zhuan Zhou Lung-Sen Kao
17	2013	Rouen (France)	Youssef Anouar
18	2015	Cairns (Australia)	Frederic Meunier Damine Keating
19	2017	Sheffield (UK)	Elizabeth Seward
20	2020	Chennai (India)	Nitish Mahapatra
21	2022	Hamburg (Germany)	Manfred Lindau

## Biology of the chromaffin cell and the adrenal medulla: state-of-the-art before the ISCCB meetings begun

**Fig. 1:**
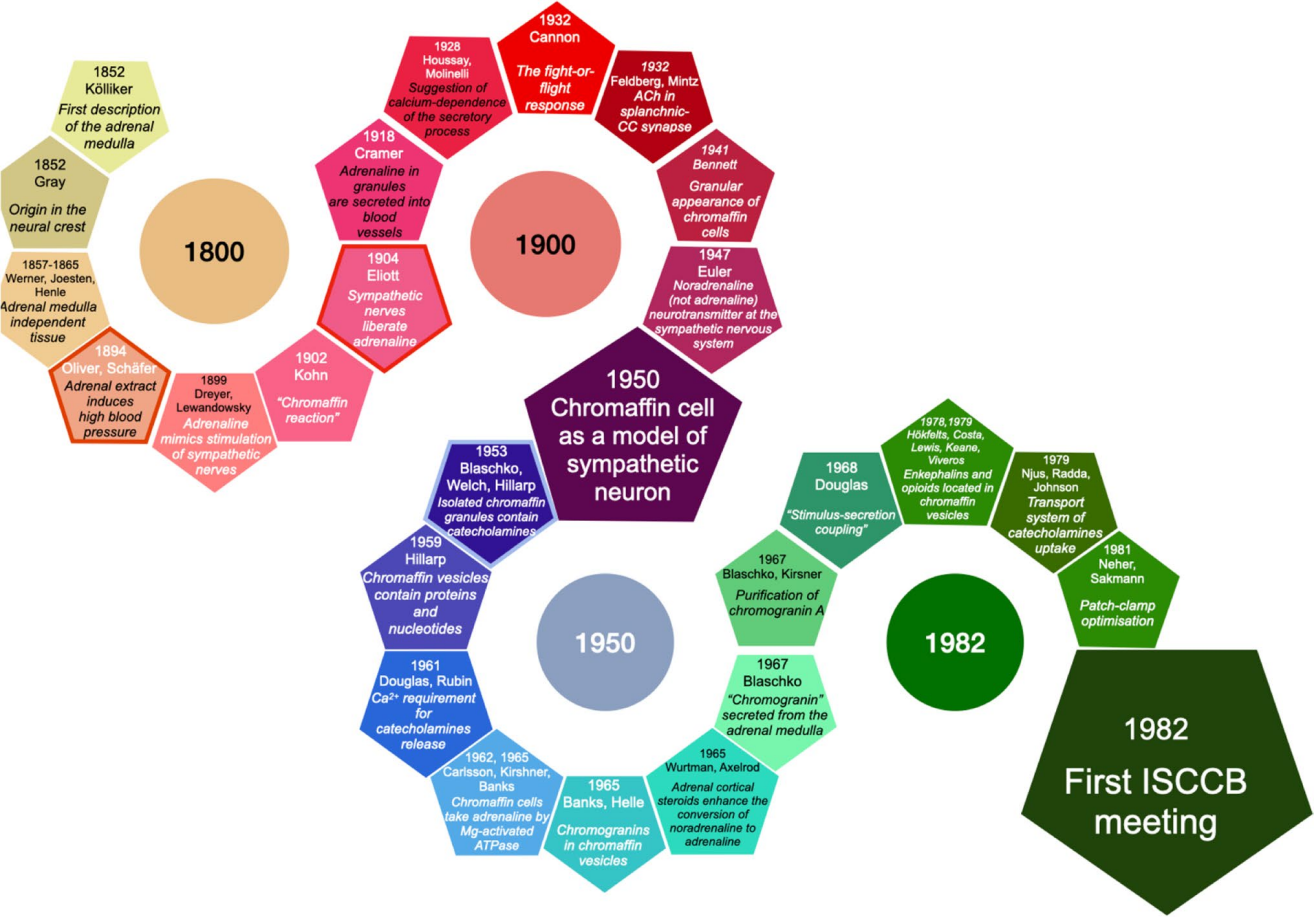
Main hints accumulated along the nineteenth and twentieth centuries, on structural, biochemical and functional aspects of the adrenal medulla and its chromaffin cells

During the first historical era, the concepts of chromaffin cell biology mainly focused anatomical, structural, biochemical, and some functional aspects of the adrenal medulla and CCs. Stephen W. Carmichael (Mayo Clinic, Rochester, USA) and Hans Winkler (Innsbruck, Austria), who contributed much to our understanding of the biology of the adrenal medulla and chromaffin cells, wrote a classical review concerning the history of this sympathetic-like organ [31]. They gave an account of the knowledge accumulated particularly along the nineteenth and twentieth centuries, on structural, biochemical, and functional aspects of the adrenal medulla and its chromaffin cells (CCs). A summary of the main historical hints is documented in Fig. 1.

In 1852, Albert von Kölliker first described the fine structure and function of the adrenal medulla, which was clearly different from the cortex. He stated: “I consider the cortical and medullary substances as physiologically distinct”. And added: “The cortex may, provisionally, be placed with the so-called blood–vascular glands, and a relation to secretion assigned to it; while the medulla, on account of its extremely abundant supply of nerves, must be regarded as an apparatus appertaining to the nervous system, in which the cellular elements and the nervous plexus either exert the same reciprocal action as they do in the grey nerve-substance, or stand in a relation as yet wholly unascertained, towards each other” [95].

The identity of adrenal medullary tissue, as an organ independent from the cortex, derived from a histochemical reaction to chromate salts as a brownish deposit [76, 87, 191]. The term “chromaffin reaction” was applied to cells that reacted in this manner to chromium, the adrenal medulla being the prime example. The chrome reaction was found to be a reduction by adrenaline [147]. The terms “chromaffin reaction” and “chromaffin cell”, were so named by Alfred Kohn [92]. A step further in understanding the structure of the adrenal medulla was related to the observation that osmic acid vapor demonstrated “adrenaline granules” as “giving the appearance of fine coal dust scattered over the medulla” [40]. Cramer linked this image to an increase in secretory activity due to the passage of the osmiophilic granules into the blood vessels; thus, this observation described for the first time the essential event in the secretion of catecholamines from the adrenal medulla.

The embryological origin of the adrenal medulla was initially studied at the middle of the nineteenth century; it was suggested that it originated in association with sympathetic ganglia [153]. A few years later, Henry Gray reported that the adrenals arose from “two grayish white masses, which lie one on each side of the aorta, between this tube and the upper and inner extremity of the Wolffian bodies, and are perfectly separate” [67]. These early observations were scarcely improved until the classic studies of chimeras by Nicole Marthe Le Douarin in the 1970s, on the migration and differentiation of neuroblasts at the autonomic nervous system and neuroectodermal mesenchymal derivatives [105].

By the end of the nineteenth century, the adrenal gland’s basic morphology, histochemistry, and embryology were known. Thus, the stage was set for the breakthrough experiment of George Oliver and Edward Schäfer on the injection of an adrenal extract that caused a sharp increase in the dog’s blood pressure [148]. This experiment was heralded as the first demonstration of a hormonal effect, a milestone in endocrinology.

A few years later, George P. Dreyer and M. Lewandowsky noted the correspondence between the effects of administered adrenaline and the effects of stimulation of postganglionic sympathetic nerves [53, 109]. This was confirmed by a student of John N. Langley, Thomas Renton Elliott, who in 1904 convincingly suggested that the sympathetic nerves produced their effects by liberating adrenaline [55]. This was the beginning of the concept of neurochemical transmission. In 1946, Ulf von Euler showed that the neurotransmitter of the sympathetic nervous system was noradrenaline [58].

Another breakthrough was the discovery of chromaffin granules based on observations under the light microscope of the granular appearance of CCs [11]. In 1953, Hermann Blaschko and Arnold Welch on the one hand, and Nils-Ake Hillarp on the other demonstrated that centrifugation of homogenates of the adrenal medulla led to sedimentation of the chromaffin granules that contained the major part of catecholamines [17, 79]. This was the first time a secretory organelle was isolated, paving the way for the discovery of hormone- and neurotransmitter-containing organelles in other organs. Hence, the first-discovered secretory organelle of the adrenal medulla remained the best characterized and has often served as a “model” organelle [193]. The classical book by Richard Coupland on “The natural history of the chromaffin cell” provides a detailed account of the morphology of chromaffin cells and chromaffin vesicles, using electron microscopic approaches [37].

From the 1950s onward, the emphasis on the adrenal medulla work shifted from the study of adrenal medullary function per se to an outlook where the adrenal CC was regarded as a model neuron in general, and as a model sympathetic neuron in particular. The uptake of compounds into chromaffin granules (CGs) was a matter of study in the 1960s. In the presence of magnesium ions and ATP, it was observed that CGs took up adrenaline against a concentration gradient [29, 94]. This was mediated by a magnesium-activated ATPase of the CG membrane [8]. Ten years later, two independent groups demonstrated this to be a coupled transport system: the ATPase actively drives protons into the CG and the pH and electrical proton gradients across the CG membrane drive catecholamine uptake [89, 142].

Much work was devoted to the elucidation of the composition of the CG, a model of a neurotransmitter-containing vesicle. For instance, in 1959, the work of Hillarp demonstrated that proteins and nucleotides are contained within CGs [78, 80]. Later on, Karen B. Helle first characterized the soluble proteins of CGs and named then as chromogranins; using antibodies against such peptides, the first biochemical demonstration for exocytosis was achieved as no other mechanism could explain the release of a large protein together with catecholamines [9]. Soon thereafter, Blaschko’s group had also given the name chromogranin to a protein within CGs, and also showed that it was secreted from the adrenal medulla [16]. The same year, 1967, Hans Winkler working in Blaschko’s laboratory and independently Norman Kirshner, purified chromogranin A [169, 171].

The CG cocktail of proteins, nucleotides, and catecholamines was enriched by enkephalins, first identified by Thomas G.M. Hökfelts’s group [166]. Within only a year, studies in the laboratories of Humberto Viveros, Sidney Udenfriend and Erminio Costa demonstrated that enkephalins and/or opioid peptides were located within CGs [36, 110, 189]. Of interest is to point out that the adrenal medulla is one of the richest sources of enkephalins in mammalians. Therefore, this tissue was used extensively for opiate studies including the simultaneous elucidation of pre-proenkephalin protein by three independent groups [35, 69, 143].

An early study suggested the Ca^2+^-dependence of the secretory process [81]. However, in the 1960s, a more direct evidence was provided by elegant classical experiments done in the laboratory of William W. Douglas. They demonstrated that Ca^2+^ was the only ion necessary to trigger the explosive release of catecholamines from the perfused cat adrenal gland stimulated with acetylcholine (ACh) [52], the physiological neurotransmitter at the splanchnic nerve-CC synapse [59]. Furthermore, augmented ^45^Ca^2+^ entry into adrenal medullary CCs stimulated with ACh was also demonstrated in Douglas’ laboratory [51]. On the basis of these and other experiments, and extrapolating the concept from the excitation–contraction coupling process in muscle, Bill Douglas coined the term stimulus-secretion coupling [50]; in this process, it could be predicted that Ca^2+^ entry through voltage-dependent Ca^2+^ channels (Cav) could elicit an increase of the cytosolic Ca^2+^ concentration that was responsible for triggering exocytosis by, at that time, an unknown mechanism.

As commented above, the adrenal gland comprises two distinct organs in their morphology, physiology, embryology, and function namely the cortex and the medulla. As they are so close, the question of whether they crosstalked arose. Two observations partially answered this question. On the one hand, Richard Jay Wurtman and Julius Axelrod found that adrenal cortical steroids enhanced the conversion of noradrenaline into adrenaline [197]. On the other hand, José María Trifaró and Klaus Unsicker separately observed that when adrenal CCs in culture were exposed to nerve growth factor (NGF), they extended neurite-like processes; this effect was inhibited by glucocorticoids [185, 187].

All these findings have led to the idea that the adrenal medulla CC is a good neuron model. The extraordinary knowledge accumulated in the period 1980–2022 in which the 21 meetings of ISCCB have been celebrated, thanks to high resolution and super-resolution imaging, electrophysiological and molecular biology techniques, add solid evidence on the CC behaving as a neuron-like cell.

## The origin of the ISCCB meetings

For years before the ISCCB meetings began, huge international conferences on the natural catecholamines (dopamine, noradrenaline, and adrenaline), acting as neurotransmitters or hormones both centrally and peripherally, were being celebrated. In the large scientific programs of those congresses, the adrenal medulla and its CCs were poorly represented. The first meeting, mainly focusing on structural and functional aspects of CCs and the adrenal medulla, was held in the beautiful Hakone National Park in Japan, in July 1981. It was a satellite symposium of the huge Congress of IUPHAR (International Union of Pharmacology), held in Tokyo at that time. The Hakone meeting was entitled “Synthesis, storage, and secretion of adrenal catecholamine: dynamic integration of functions” and was organized by F. Izumi, K. Kumakura, J. Kurosawa, and M. Oka. E. Costa, C. Gagnon, H.B. Pollard, M. Sandler, and O.H. Viveros helped to shape the scientific program. Thus, in Hakone, one of the authors of this review (AGG) had the first contact with probably the first “independent” international meeting entirely devoted to the adrenal CC. Since this initiative was precious, the Hakone meeting appeared to be only an isolated experience, a mere satellite to IUPHAR. However, this experience probably contributed to the initiative of organizing a series of ISCCB meetings that emerged in Nottingham, UK.

At those times, the International Society of Neurochemistry (ISN) was well established. In the programs of ISN meetings, the topics on biochemical aspects of the synthesis, storage and metabolism of adrenal catecholamines, as well as those related to their exocytotic release from adrenal glands and isolated CCs were often included. The eighth meeting of ISN was held at Nottingham, UK in September 1981, only a few months after the Hakone meeting. Some of the scientists who attended at that meeting were also present at the ISN meeting. Bruce Livett organized an ambitious round table on “The chromaffin cell as a neurochemical model,” with 12 speakers: Bruce Livett, O. Humberto Viveros, Derek E. Knight, José María Trifaró, Dominique Aunis, Simon Lemaire, Klaus Unsicker, Antonio G. García, Hans Thoenen, Gordon Guroff, Elisabeth Fenwick, and Erminio Costa.

In this multidisciplinary session, the described procedures available for isolating and maintaining CCs in primary cultures for several days were discussed; particular attention deserved the criteria for assessing purity, yield, identity, and survival of CCs in culture. Of special interest was the culture of bovine adrenal CCs, optimized by Bruce Livett, with an almost unlimited number of CCs that could be prepared from glands easily obtained in a local slaughterhouse. The cultured bovine CCs greatly increased the interest of many groups in the use of these highly homogeneous cell preparations to investigate different questions linked to ion channels, neurochemistry of proteins, Ca^2+^ ion fluxes, and exocytosis. In fact, in the round table at Nottingham, some studies on the role of nerve growth factor (NGF) and substrate adhesion in neurite outgrowth from cells were discussed. Also, the role of ATP and contractile proteins in the secretory process were also commented. Of interest was the proposed role of neuropeptides (i.e., substance P, somatostatin, and the enkephalins) as neuromodulators of catecholamine secretion.

Hence, the main outcome of the round table at Nottingham was the idea that large-scale isolation and maintenance of two cell types of neural crest origin namely, adrenal CCs (adrenal paraneurons) and pheochromocytoma cells (PC12), which were described in 1976 by Lloyd Green and Art Tischler [68], could be easily obtained. The combination of these two models offered several advantages over the complex mixed cells of the brain, for neurochemical and pharmacological studies on neurite outgrowth and the exocytotic release of neurotransmitters and hormones. During a visit to Chatsworth House organized by ISN, Hans Winkler (Austria), Dominique Aunis (France), Humberto Viveros (USA), Bruce Livett (Australia), José María Trifaró (Canada), and Antonio G. García (Spain) discussed the idea of regularly organizing a symposium on the adrenal medulla and its CCs. We agreed to organize the first in Ibiza, one of the Balearic Islands of Spain, at the Mediterranean Sea. So, on the bus that drove us back to Nottingham, we wrote a draft program for the first ISCCB Symposium of 1982 in Ibiza. Thus, the first Scientific Committee, for the next 40 years of CC biology symposiums around the world, was born in a bus crossing the forests of Nottingham, where Robin Hood had his adventures.

## The first ISCCB meeting at Ibiza

The first meeting on CCs was held at Ibiza in September 1982, 1 year after the Hakone and the Nottingham meetings. It was organized by Antonio García and Valentín Ceña together with other members of the Pharmacology Department of the Medical School (Universidad Autónoma de Madrid). It was attended by 97 scientists from Europe, the USA, Canada, Israel, and Japan. The first international program committee was formed by D. Aunis, A.G. García, M. Oka, J.H. Phillips, J.M. Trifaró, O.H. Viveros, E.W. Westhead, and H. Winkler. It was agreed that a speaker would be invited for each major group working in the field and that the program should not be overloaded with too many scientific activities. So, 30–35 talks (25-min plus 5-min discussions) were scheduled for 4 days (Monday, Tuesday, Thursday, and Friday); Wednesday was left entirely for cultural and social events.

Ample free time was allowed to see the posters, to favor the exchange of ideas, to stimulate friendship and contact among people, and to promote the establishment of collaborations between different laboratories. Wednesday was free for a 1-day excursion to the almost wild island of Formentera, where we enjoyed a gigantic paella and had a lot of fun visiting the island and swimming in the Mediterranean Sea.

At Ibiza, the Program Committee agreed that a scientist in charge of organizing a meeting would serve for the next ISCCB symposia. The idea was to facilitate the recruitment of a wide range of scientists, including younger researchers who were building up their own groups.

The Ibiza ISCCB-1 meeting created a tradition, the “spirit of Ibiza,” that was grounded in the following ingredients: (1) to hold the meetings in an isolated place with a relaxed atmosphere that would encourage interactions among scientists; in fact, the Ibiza meeting was held at the fantastic Hotel Hacienda, located on a top of a hill, looking at the Mediterranean Sea; (2) to build a program leaving enough time for discussion around the posters, meals, and cultural events; (3) this will contribute to create an atmosphere of friendship to promote collaborations between different laboratories. At Ibiza, the Program Committee supported the offer of Dominique Aunis to organize the second ISCCB meeting in Colmar, France, in 1984. We will next discuss the main hints of the evolution of our present ideas on the adrenal medulla and its CC structure and function, along the last 40 years of ISCCB meetings around the world. The summary of the Ibiza meeting was done by 11 senior scientists that in 5-min presentations, tried to predict the direction of future research on the topics presented in Table 2. We will approximately follow this scheme to define the accumulated new knowledge on each topic, during the 40 years of ISCCB meetings.

**Table 2:** Perspectives for the evolution of various themes on chromaffin cell biology commented by experts attending the Ibiza meeting in 1982

1	Development and plasticity Migration of neural crest cells New neurotrophic factors **Rita Levi-Montalcini (Italy)**
2	Catecholamine synthetic enzymes 3D-structure Gene regulation **Norman Weiner (USA)**
3	Monoamine transporters Structure Gene regulation **Jean Pierre Henry (France)**
4	Chromaffin granules New components of its core structure **Hans Winkler (Austria)**
5	Co-messengers Opiates New regulatory peptides Chromogranins and secretogranins: gene regulation **Bruce G. Livett (Australia)**
6	Ion channels Exocytosis **Peter Baker (UK)**
7	Autoreceptors and exocytosis **Lennart Stjärne (Sweden)**
8	The exocytotic cycle Fate of vesicles Vesicle sorting and retrieval **Jean Jacques Nordman (UK)**
9	Cytoskeleton F-actin, scinderin Vesicle transport Vesicle pools **José María Trifaró (Canadá)**
10	The exocytotic machinery **Harvey B. Pollard (USA)**
11	Membrane fusion The fusion pore **Werner de Potter (Belgium)**

## The biology of the adrenal medulla and its chromaffin cells: evolution of our knowledge along the 40 years of ISCCB meetings

Several reviews have approached the evolution of physiological molecular and pharmacological aspects of the sympathoadrenal axis and the adrenal medulla and its CCs. The most recent comprehensive review on this topic has been written by Emilio Carbone (Italy), Lee Eiden (USA), Ricardo Borges (Spain), Antonio G García (Spain), and Arturo Hernández-Cruz (Mexico); this extensive review has been recently published and provides a detailed overview on the Ibiza’s questions, with 692 references [28]. In this context, it makes no sense to repeat here such exhaustive information. We will, therefore, succinctly highlight the main advances in our knowledge, following the questions raised at the first ISCCB meeting, 40 years ago. Additionally, we here add a question on CC and disease. This will necessarily leave aside many topics, information on which can be found in the abovementioned review as well as in other previous reviews mainly focusing Ca^2+^ channels, cell excitability, Ca^2+^ signaling, exocytosis, and endocytosis [48, 63, 64, 119].

### Development, plasticity, and neurotrophic factors

At Ibiza, Rita Levi-Montalcini commented on two critical experiments that demonstrated the ability of rat CCs to evolve into a sympathetic neuron phenotype. One of these experiments was performed by Klaus Unsicker et al., [187] at Johns Hopkins University; they found that immature CCs from the rat adrenal medulla, cultured in the presence of nerve growth factor (NGF), acquired the biochemical and morphological properties of sympathetic neurons. In the second experiment done at the Italian Council for Research (CNR) in Rome, Aloe and Levi-Montalcini demonstrated in vivo that the application of NGF into the rat fetus and continued for 3 weeks after birth induced the differentiation of CCs into sympathetic neurons within the adrenal gland [5].

Of interest is the observation that several proteins from chromaffin granules promote the survival of neurons thus behaving as neurotrophins [100]. On the other hand, the discovery of neural-crest-derived progenitor cells in the adult adrenal medulla [54] raised the issue of their potential use in regenerative treatment of neurodegenerative diseases [20]. Growth factors and peptides with growth factor-like efficacies have been identified in CCs, i.e., fibroblast growth factors (FGFs), transforming growth factor-B1 (TGF-B1), and interleukins [186]. The release from CCs of these bioactive molecules may have regulatory functions in cell proliferation and differentiation.

An interesting recent review [10] highlights the development of the adrenal medulla and its interaction with cortical cells. Of interest is the observation that adrenal chromaffin-like cells have been generated from human-induced pluripotent stem cells [3]. These strategies may potentially help to gain further insights into the maintenance and mechanism of CC development in the adult adrenal. In addition, such cells-organoids can potentially treat neurodegenerative diseases [21].

### Catecholamine synthetic enzymes

The biosynthesis of catecholamines (adrenaline mainly in adrenal medullary CCs and noradrenaline mainly in sympathetic nerve terminals) is a complex and tightly controlled neurochemical process that ensures the storage and secretion of adrenaline in response to stress. Hermann Blaschko predicted the pathway involved. Since then, this pathway has been extensively studied, and the various elements of the underlying molecular machinery have been identified and characterized, as recently reviewed [12]. It involves the sequential activity of four enzymes: tyrosine hydroxylase (TH), aromatic L-amino acid decarboxylase (AADC), dopamine β-hydroxylase (DBH), and phenylethanolamine-N-methyl transferase (PNMT). DBH is located at the chromaffin granule, where it catalyzes the conversion of dopamine into noradrenaline. The other three enzymes are in the cytosol: TH catalyzes the conversion of L-tyrosine into L-DOPA, AADC converts L-DOPA into dopamine, and PNMT catalyzes the conversion of noradrenaline into adrenaline. The rate limiting step in catecholamine synthesis rests in TH, with multiple models of regulation [41]. This complexity stimulated great interest in many fields of biochemical research, including some pathologies. For instance, several TH polymorphisms in the general population appear to be associated with increased circulating levels of noradrenaline and hypertension [107, 152].

DBH catalyzes the conversion of dopamine into noradrenaline inside chromaffin granules; thus, dopamine that has been synthesized in the cytosol must first enter the chromaffin granule through vesicular monoamine transporters (VMATs). Finally, PNMT methylates noradrenaline to adrenaline; this enzyme expression is controlled by glucocorticoids as adrenal medullary CCs are exposed to high concentrations of these hormones, secreted from nearby cortical cells [201].

Increased levels of mRNAs encoding catecholamine-synthetizing enzymes occur in response to stress. In adrenal CCs, this response is rapid, especially for TH and PNMT [163]. DBH gene expression occurs upon prolonged or repeated stressors [102, 184].

### Monoamine transporters

The first description of ATP-dependent catecholamine uptake into storage chromaffin granules was given by Norman Kirshner [94] and, independently, by Arvid Carlsson et al., [30]. A breakthrough in the understanding of the bioenergetics of the transport was the proposition that monoamine uptake was indirectly coupled to ATP hydrolysis [90, 141]. This ATPase is a proton pump [140] that exchanges one cationic catecholamine for two protons. Thus, the intragranular catecholamine concentration has been measured as 800–1000 mM [4, 130], whereas the cytoplasmic concentration has been measured 10–100 µM [132]. By crossing the granule membrane, cationic lipophilic amphetamines dissipate the pH gradient; this alkalinization of the granular matrix leads to rapid efflux of catecholamines [179].

For physiological reasons, the presence of an exchange reaction is a relevant issue: in adrenergic cells, noradrenaline has to diffuse out of the granule to be methylated in the cytoplasm; thus, a transport mechanism exchanging noradrenaline and adrenaline would save energy. An elegant approach using cyclic voltammetry has confirmed that in adrenergic cells, noradrenergic granules were extremely rare; this was surely due to the high efficiency of noradrenaline methylation by cytosolic PNMT, and adrenaline uptake was high since most of these CCs (75%) contained granules with only adrenaline [34].

Pharmacologically, various drugs are excellent ligands for the monoamine transporter of chromaffin granules, i.e., tetrabenazine, ketanserin, and reserpine. They have been good tools for characterizing the kinetics of the transporter (see the excellent review of Henry et al., [77].

### The chromaffin granule: core components and structure

Chromaffin granules are, essentially, the same organelles as the large dense core vesicles (LDCVs) found in many neuroendocrine cells and in some neurons. As commented in “The biology of the adrenal medulla and its chromaffin cells: state of the art before the ISCCB meetings” section, the storage of catecholamines in CGs was pioneered by biochemists [17, 79] and morphologists [38, 108]. An early review described the biochemical cocktail of CGs [192]. Small molecules such as catecholamines are accompanied by nucleotides, including diadenosine polyphosphates [158], ascorbic acid, and calcium. In addition, neuropeptides as enkephalins were shown to be stored and co-released with catecholamines [190], as well as neuropeptide Y (NPY).

#### Chromogranins

Chromogranins (Cgs) are the main protein components of CGs. The most important are chromogranin A (CgA) and chromogranin B (CgB) and, to a lesser extent, secretogranin II (SgII). To date, four main physiological roles have been attributed to Cgs [144]:

1. To act as a source of biologically active peptides. These granins are secreted during regulated exocytosis, and they may fulfill hormonal, autocrine, and paracrine activities through their peptide derivatives [72, 129, 181, 200].
2. To promote granule biogenesis. Thus, the downregulation of CgA [93] and CgB [83] provokes a loss of secretory granules in PC12 cells, while its overexpression induces the biogenesis of structures resembling secretory granules. Indeed, these granule-like structures can release/secrete their contents [13, 83, 93, 175]. However, secretory granules can form independently of CgA expression [42, 75, 118, 131], consistent with findings in CgB knockout mice [47, 146]. Indeed, granule biogenesis and Ca^2+^ -evoked secretory responses are both evident in CCs from Cgs-KO animals [46, 47].
3. To act as chaperones for prohormone-mediated sorting and packaging of neuropeptides in granules within the trans-Golgi network [39, 128, 137].
4. To facilitate the storage of catecholamines and ATP [56, 74, 130, 135]. This was the first function attributed to Cgs being currently considered to exhibit high-capacity and low-affinity buffers. Similar interactions with soluble species such as catecholamines and ATP are also likely to occur forming a matrix, which probably corresponds to the electron-dense core observed in electron microscopy images.

The ability of secretory vesicles to actively accumulate enormous concentrations of solutes has intrigued scientists for decades. H^+^ is an important component of vesicles, and it is concentrated by a specific V-ATPase to maintain an inner pH of 5.5, approximately coinciding with the isoelectric point of Cgs. As the association of Cgs with other solutes is pH-dependent [74], vesicular pH may also regulate the ability of CgA to form aggregates [182], thereby playing a functional role in the dynamics of vesicular Ca^2+^, ATP, and catecholamines.

CgA was described in the mid-sixties [16] as the first of a series of acidic proteins known as granins, of which nine members have been identified to date [19, 181]. Chromogranins are characterized by highly hydrophilic and acidic primary amino acid sequences [84], as well as the presence of multiple paired basic residues that form cleavage sites in pro-hormones to generate bioactive peptides [73, 106]. CgA and CgB share a tendency to self-aggregate at acidic pH values and high Ca^2+^ concentrations, conditions typical of the lumen of the trans-Golgi network and secretory granules [84, 160, 181, 194]. Aggregated granins provide the physical driving force to induce the budding of trans-Golgi network membranes, resulting in the formation of dense core granules [97, 98].

Cgs-KO mice have been developed using distinct strategies [118, 146]. By crossbreeding these two strains, the double CgA/B-KO mouse was created being viable and fertile in homozygosis [47]. In the partial or total absence of Cgs, the quantum size and the kinetics of exocytosis is largely affected. Indeed, Cgs are responsible for about 50% of the total capacity of granules to accumulate catecholamines [47]. Nevertheless, 400–500 mM of catecholamines (half of the content in wild-type animals) is still an enormous concentration. In other words, another intravesicular component responsible for this accumulation seems to operate in this process.

#### ATP, the necessary intravesicular component

Virtually, most types of secretory vesicles found in cells contain ATP, which often accumulates at high concentrations and commonly, in conjunction with different types of neurotransmitters. However, the reason for this widespread distribution of ATP remains a mystery. Although ATP is present in all animal species, including primitive life forms like *Giardia lamblia* that lack Golgi complexes and mitochondria, detecting of ATP in the secretory vesicles of sympathetic neurons was the first example of co-transmission [23]. However, given the ubiquitous accumulation of ATP in secretory vesicles, it might be considered that it is the other neurotransmitters that coincide with ATP rather than the other way around [18]. Indeed, perhaps ATP should be regarded as the first molecule used as a transmitter in primitive live forms.

Besides its role as the “energy coin,” ATP possesses the ability to bind catecholamines. This interaction results in a reduction of the osmotic forces as was initially proposed by Edward Westhead [96]. The physical association of ATP with catecholamines was also recently demonstrated using electrochemical in vitro approaches [180] and in living chromaffin cells [56].

#### Calcium

The intragranular concentration of calcium has been measured as 40 mM using targeted aequorins. Therefore, and considering that the granular component occupies ≈17% of the cell volume, granules are, by far, the main Ca^2+^ storage organelles in chromaffin cells [198]. However, most of this Ca^2+^ seem to be chelated with other intravesicular components [164] leaving 40–50 µM of free Ca^2+^.

The possible role of vesicular Ca^2+^ in the aggregation of soluble products within the secretory vesicle has been proposed. As divalent cations promote the shrinkage of the granule matrix, being the two of the major chemicals necessary for exocytosis (ATP and Ca.^2+^) highly concentrated in chromaffin granules, the attractive idea of their contribution to granule motion and exocytosis has been proposed [25, 133]

### Ion channels and exocytosis

**Fig. 2:**
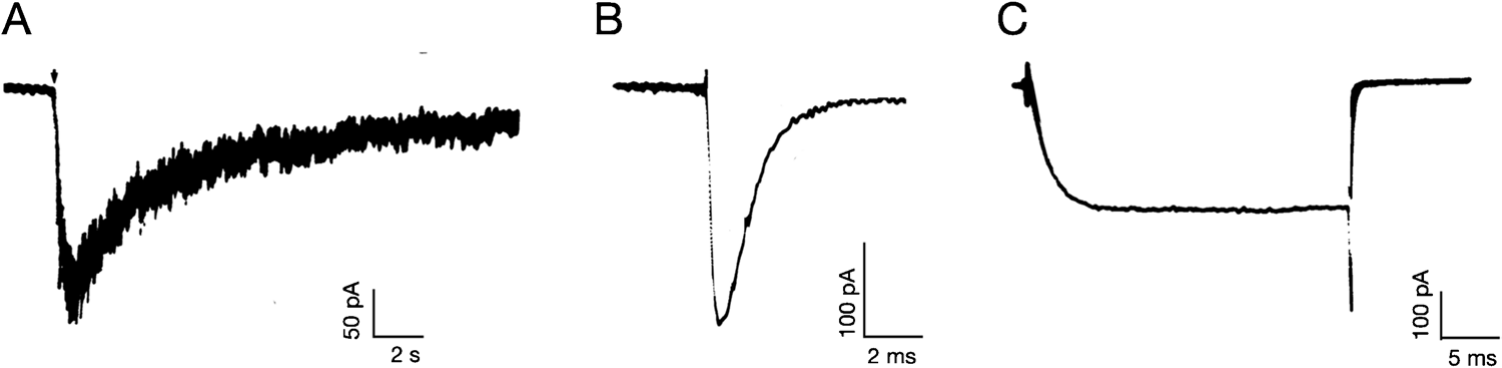
Examples of whole-cell ion currents through (**A**) nACh, (**B**) Na.^+^ channels, and (**C**) Cav channels measured in voltage-clamped bovine CCs using whole-cell configuration of the patch-clamp technique. Adapted from Fenwick et al. [60, 61]

When the ISCCB meetings commenced at the beginning of the 1980s, the laboratories of Erwin Neher and Bert Sackmann published their classical paper on the improved patch-clamp technique [71]. This gave rise to an explosion of studies on the characterization and biophysical properties of a myriad of ion channels in many excitable cells, including CCs. In fact, the first reports on whole-cell nicotinic receptors currents and acetylcholine (ACh) evoked action potentials [60] as well as in sodium and calcium currents [61] in bovine CCs, came from the laboratory of Erwin Neher (Fig. 2).

In the 1990s, the patch-clamp techniques and the discovery of various marine toxins at the laboratory of Baldomero Olivera [149] served as two powerful tools to map the great variety of ion channels expressed by CCs. In the extensive review mentioned above, the pallet of Na^+^, Ca^2+^, and K^+^ channels expressed by CCs is commented in detail [28]. This abundance of ion channels appears excessive for CCs that are a “relay element” that passively follow the neural input generated at the CNS. However, several studies carried out in the laboratory of Emilio Carbone suggest an explanation for this rich ion channel palette: they can also sustain an intrinsic electrical activity of CCs that, under humoral conditions, may contribute to characteristic forms of catecholamine release [112, 121].

**Fig. 3:**
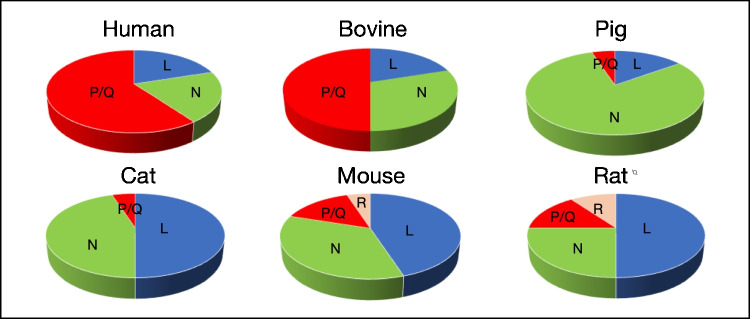
Relative densities of high-voltage activated (HVA) calcium channels in different mammalian species (modified from [64]). L, Cav1 channels, P/Q Cav2.1 channels, N, Cav 2.2 channels, R Cav2.3 channels

Practically, all voltage-activated calcium channels (Cav1, 2, 3) expressed by neurons have also been found to be functionally expressed in CCs from six mammalian species, including humans. The surprising finding was that those channel subtypes were defined at different densities in those six animal species (Fig. 3).

This asymmetric expression of Cav subtypes has a functional correlate in the Ca^2+^-dependent exocytotic response, triggered by the depolarization of CCs from the six mammalian species. For instance, the Ca^2+^entering the cell through slow-inactivating L-channels dominates the secretory response in cat, mouse, and rat CCs; in contrast, in bovine and human CCs, the entry of Ca^2+^ through P/Q channels dominate the secretory process. Additionally, depending on the type of depolarizing stimulus used, one or other Cav channel controls the secretory process [64].

At least four questions remain unanswered on the functional role of such a variety of Cav channels expressed by CCs: (1st) Why does a simple cell lacking dendrites and axons require various Cav channel subtypes?; (2nd) Do all Cav channels contribute to exocytosis?; (3rd) Are those channels contributing more to exocytosis colocalized with the secretory machinery more tightly than other channel types?; and (4th) Do some of the channel subtypes contribute to functional aspects of CCs beyond exocytosis? A curious finding supported the specialization of Cav subtypes to regulate such function: The L subtype selectively controls the Ca^2+^-dependent endocytosis occurring after a depolarizing stimulus in voltage-clamped bovine CCs (see review by Nanclares et al., and Rosa et al., [136, 159] for a more detailed explanation on Cav channels and endocytosis). Of interest are also the experiments suggesting that L-type channels control the spontaneous firing of action potentials in rat and mouse CCs (see Carbone et al*.*, [28] for a detailed explanation.

Another interesting question is related to the complexities of the mechanisms involved in the fine-tuning of Ca^2+^ handling by CCs. So, the physiological stimulation of CCs with ACh triggers the release of catecholamines by a Ca^2+^-dependent process [52]. To trigger secretion Ca^2+^ has to enter CCs through Cav channels, as initially suggested by the enhanced radiolabeled Ca^2+^ uptake into CCs stimulated with ACh [51]. With the discovery of the patch-clamp technique, the first whole-cell Ca^2+^ currents were recorded in voltage-clamped bovine CCs [61]. This simple, early pathway was later complicated when it was discovered that the Ca^2+^ entering through Cav channels upon cell depolarization gets redistributed in two cell organelles namely, the endoplasmic reticulum (ER) and mitochondria [63, 64].

**Fig. 4:**
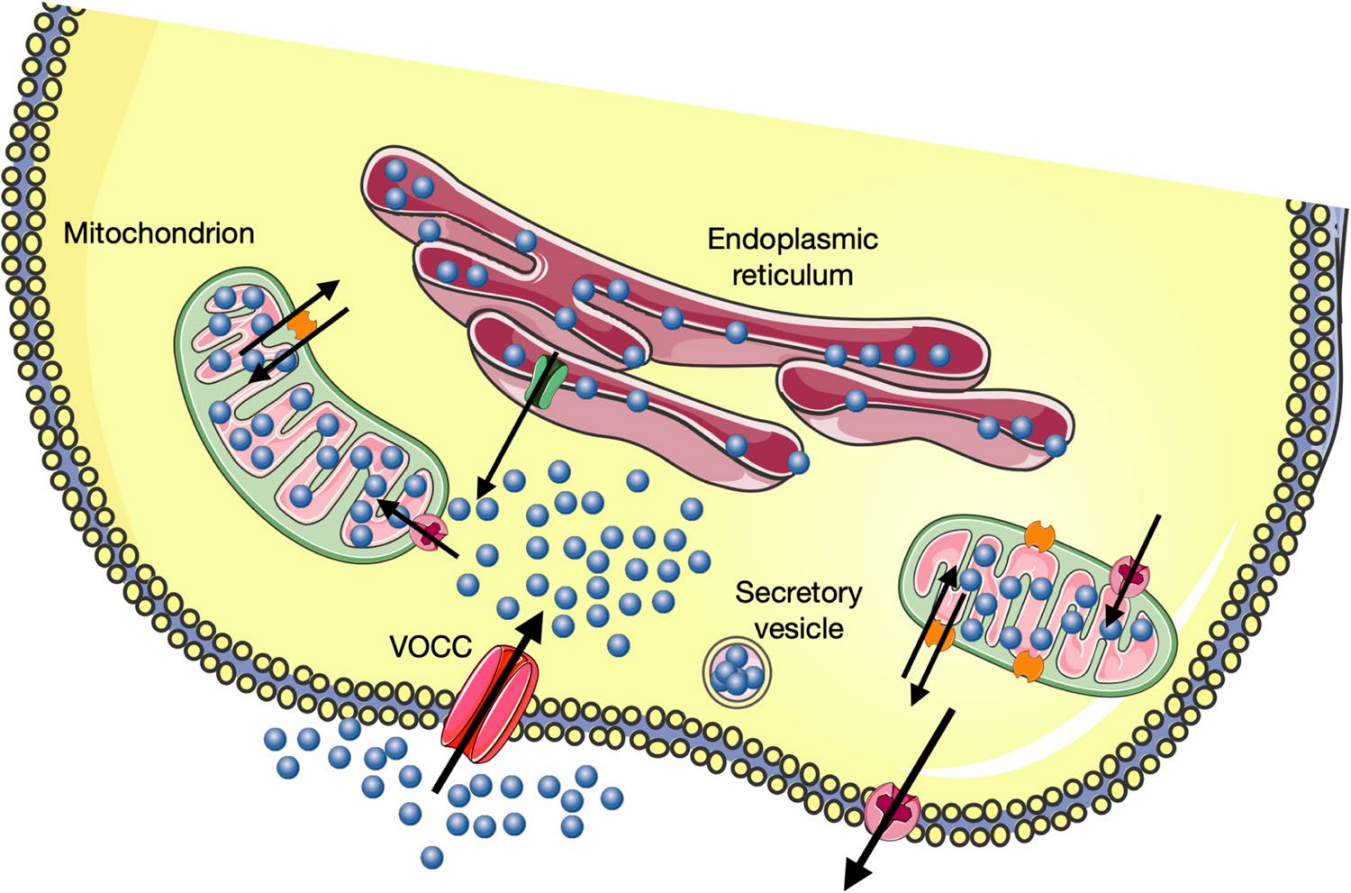
The hypothesis of a functional triad to control the dynamics of Ca^2+^ in CCs. Cav, Ca^2+^ entry through voltage-activated Ca^2+^ channels. Ca^2+^ that enters the cell redistributes into mitochondria and the endoplasmic reticulum. Ca^2+^ PMT, Ca^2+^ exit through plasma membrane transporters. Images obtained from Servier Medical Art, licensed under a Creative Commons Attribution 3.0 Generic License

Briefly, we simplify our view of the global Ca^2+^ handling by CCs in terms of what we refer to as a functional triad to shape the cytosolic Ca^2+^ transients ([Ca^2+^]_c_) occurring during cell activation (Fig. 4). These triads are formed by clusters of Cav channels, the ER ryanodine receptors (RyR), and the mitochondrial Ca^2+^ uniporter (MCU). Cav channels will act as the initiating signal, the RyR as the signal amplifier through the Ca^2+^ -induced- Ca^2+^ release mechanism (CICR), and mitochondria as a barrier to limit diffusion further into the cell’s core where the high-Ca^2+^ microdomains (HCMDs) generated at subplasmalemmal exocytotic sites are not required. Diffusion of Ca^2+^ from subplasmalemmal sites is achieved by the propagation of Ca^2+^ buffers and maintained by CICR and the release of Ca^2+^ at inner cytosol sites via the mitochondrial Na^+^/Ca^2+^ transporter (NCLT).

From a physiological point of view, the concept of a functional triad suggests that Ca^2+^ entry, through Cav channels and its sequestration and subsequent release by mitochondria, is central stage in the regulation of Ca^2+^ handling by CCs. The fluxes of Ca^2+^ through those channels and organelles are strongly activated during cell activation; however, they also operate during inter-stimulation periods thus contributing to maintaining Ca^2+^ homeostasis in CCs. Dysregulation of Ca^2+^ fluxes under various pathological conditions will alter the production of energy by mitochondria with the subsequent impairment of exocytosis, as discussed later.

## Frontier advances reported at the ISCCB-21 meeting in Hamburg

### Calcium channels, calcium dynamics, and exocytosis

During the 1990s and 2000s decades, studies on voltage-gated Ca^2+^ channels (Cav) and Ca^2+^ dynamics in CCs were hot topics at ISCCB meetings. However, in the ISSCB-21 meeting at Hamburg, only a few communications on this topic were presented. Thus, Erwin Neher commented the seminal work of his lab on Ca^2+^ and priming dynamics comparatively in both CCs and synaptic terminals [138]. In both models, rapid bursts of release exhibited fast and slow components. However, it was puzzling that while in CCs, the amplitudes of such responses to pool-depleting stimuli very much depended on the level of cytoplasmic Ca^2+^ concentration ([Ca^2+^]_c_) before the stimulus; at synapses, the responses seemed to be pretty robust independently of [Ca^2+^]_c_ levels. Thus, dynamic priming, i.e., the dependence of the readily release vesicle pool (RRP) of secretory vesicles on [Ca^2+^]_c_ levels and on the repertoire of secretory proteins, seemed to be a specialty of catecholamine release of CCs [183]. However, recent experiments suggest that synaptic plasticity may also be linked to [Ca^2+^]_c_ manipulation [120]. Furthermore, flash-and-freeze electron microscopy showed that synaptic vesicles can be observed in two states of docking at the plasma membrane, loose and tight, and that neuronal activity transiently increases the number of tightly docked synaptic vesicles [101, 139]. So, Neher concluded that in the end, work on catecholamine secretion might tell us not only about neurotransmitter response per se, but also about synaptic plasticity. An additional communication on temporal aspects of exocytotic plasticity was presented by Andrew G. Ewing. He uses a new electrochemical approach with intracellular and extracellular measurements simultaneously; this has led to time-resolved measurements of plasticity in real-time.

Another strongly Ca^2+^-dependent process is the interaction between the lipid bilayer and SNARE conformation. This strong Ca^2+^-dependent fusion efficiency on membrane order was confirmed in purified LDCVs from PC12 cells and insulin granules purified from INS1 cells, as reported by Volker Kiessling [99]. Additionally, Emely Tanguy concluded that phosphatidic acid species modulate not only Ca^2+^-dependent exocytosis but also CG transport to the cell periphery and compensatory endocytosis. Also in this regulatory aspect of Ca^2+^-dependent membrane fusion was the presentation of Yongsoo Park, concluding that cholesterol depletion abolishes Ca^2+^-dependent vesicle fusion by disrupting synaptotagmin-1-induced membrane bending; thus, cholesterol seems to be a master regulator of Ca^2+^-dependent fusion [150]. On the other hand, Frédéric A. Meunier delivered an exciting talk demonstrating that consolidation of long-term fear memory in rats is associated with a strong increase in saturated free fatty acids. He also described the impact of genetic ablation of phospholipase A1 on progressive neuromotor and cognitive decline and the disruption of free fatty acid responses, using longitudinal behavioral and lipidomic analysis over 3–12 months. Understanding this regulated lipid pathway could inspire therapeutic strategies for neurodegenerative diseases, Meunier concluded [86]. An additional communication by Liang-Wei Gong dealt with the regulation by sphingosine of synaptic vesicle exocytosis and endocytosis [85].

The dynamic organization of Cav2.1 channels was studied using single-molecule tracking to access the number and localization of individual channels over time, in synapses of hippocampal mouse neurons, as explained by Martin Heine. Light-induced crosslinking evoked transient changes in the release probability of the synapse, supporting a mobile arrangement of Cav2.1 channels within the presynaptic membrane. Another communication related to Cav channels dealt with the loss of α2δ﻿1 Ca^2+^channel subunit, which increases excitability and promotes burst firing in mouse CCs, as reported by Petronel Tuluc. Curiously, although this subunit deletion caused 40% reduction of Ca^2+^ influx, catecholamine release was increased, likely due to enhanced cell excitability induced by lesser calcium entry.

The laboratory of Emilio Carbone has contributed much to our understanding of ion channels, cell excitability, and exocytosis in CCs. At Hamburg, Emilio presented an elegant study on the extracellular recording of action potentials from rat CCs at 37 °C, using microelectrode arrays to record simultaneously from many cells. He provided evidence for two distinct spontaneous firing models (positive-going and negative-going) and well-resolved action potential-driven Na^+^, K^+^, and Ca^2+^ currents at the cell-microelectrode contact region.

### Vesicle biogenesis: the exo-endocytotic cycle

Several communications at Hamburg discussed new data on the various steps involved in LDCV biogenesis: (1st) the secretory cargo proteins composed of dense core matrix proteins and neuropeptides are produced; (2nd) they aggregate in the trans-Golgi (TGN) from which immature LDCV vesicles containing the cargo and membrane proteins such as synaptobrevin, cellubrevin, and GP III, bud off; (3rd) this newly formed LDCVs rapidly move towards the plasma membrane and further mature while loading with catecholamines; and (4th) membrane proteins can be recycled after exocytosis and appear in newly formed LDVCs within 45 min without passing through the TGN. This pathway of LDCV biogenesis has been elucidated over several decades using a large array of techniques such as pulse-chase experiments in combination with electron microscopy (EM), subcellular fractionation, and over-expression of chromogranin A labeled with fluorescent proteins. These experiments have been performed in bovine CCs and PC12 cells [57, 151, 162] and, more recently, in mouse CCs [44]. In their study, Dembla and Becherer conclude that LDCVs separate from an intermediate Golgi compartment, mature for about 1 h, and then travel to the plasma membrane. The exocytotic machinery composed of vSNAREs and synaptotagmin1, which originate from either de novo synthesis or recycling, is most likely acquired via fusion with precursor vesicles during maturation. The authors also concluded that recycling of LDCV proteins is achieved in less than 2 h. Julia von Blume delivered a very didactic talk on the mechanisms underlying the biogenesis of LDCVs, implicating chromogranins as regulators of this process, in insulin-secreting β-cells.

An early model predicted that both the LDCV membranes diffuse into the plasma membrane and, subsequently, are randomly internalized. This implied that both membranes lost their identities and that exocytosis was not directly coupled to endocytosis. However, after full-fusion exocytosis, granules and plasma membranes seem to maintain their specific protein composition. Thus, evidence for exo-endocytosis coupling came from the observation that some components of the LDCV membrane could be retrieved after exocytosis [151]. Additionally, Robert Burgoyne’s lab showed a significant augmentation in the number of coated pits that contained CG membrane components after secretagogue-stimulated CCs [66]. Based on these observations, the laboratory of Erwin Neher suggested a fast temporal coupling between exocytosis and endocytosis in bovine CCs [170].

Using EM in cultured CCs, the group of Marie-France Bader and Stéphane Gasman described the clustering of CG proteins on the plasma membrane after exocytosis [32]. This was confirmed by the group of Holz, showing that CG markers remained associated after fusion [15]. Additionally, it was also observed that those CG-bound proteins were subsequently internalized through vesicles devoid of plasma membrane markers [32]. These data argue against the idea that granule components are dispersed in the plasma membrane. Instead, they indicate that CG membranes are maintained together as “microdomains” after full-fusion exocytosis; subsequently, they are recaptured in a clathrin-dependent manner without intermixing with the plasma membrane. Further insight into the role of lipids as regulators of exo-endocytosis and the vesicle recycling pathway can be found elsewhere [14, 82].

### The exocytotic machinery at a molecular level

Twenty-five years after their discovery [165, 173], overwhelming evidence of the crucial role of SNARE (soluble NSF attachment protein receptor) complexes in many membrane fusion processes has accumulated [22, 161, 177]. SNARES syntaxin-1, SNAP-25, and synaptobrevin/VAMP2 are the core of a highly conserved, integrated molecular machine that drives synaptic transmission by synaptic vesicle exocytosis [178] and also secretion of neuropeptides, catecholamines, and neurotrophins from LDCVs [6, 188]. This topic deserved much attention at the ISSCB-21 meeting in Hamburg.

**Fig. 5:**
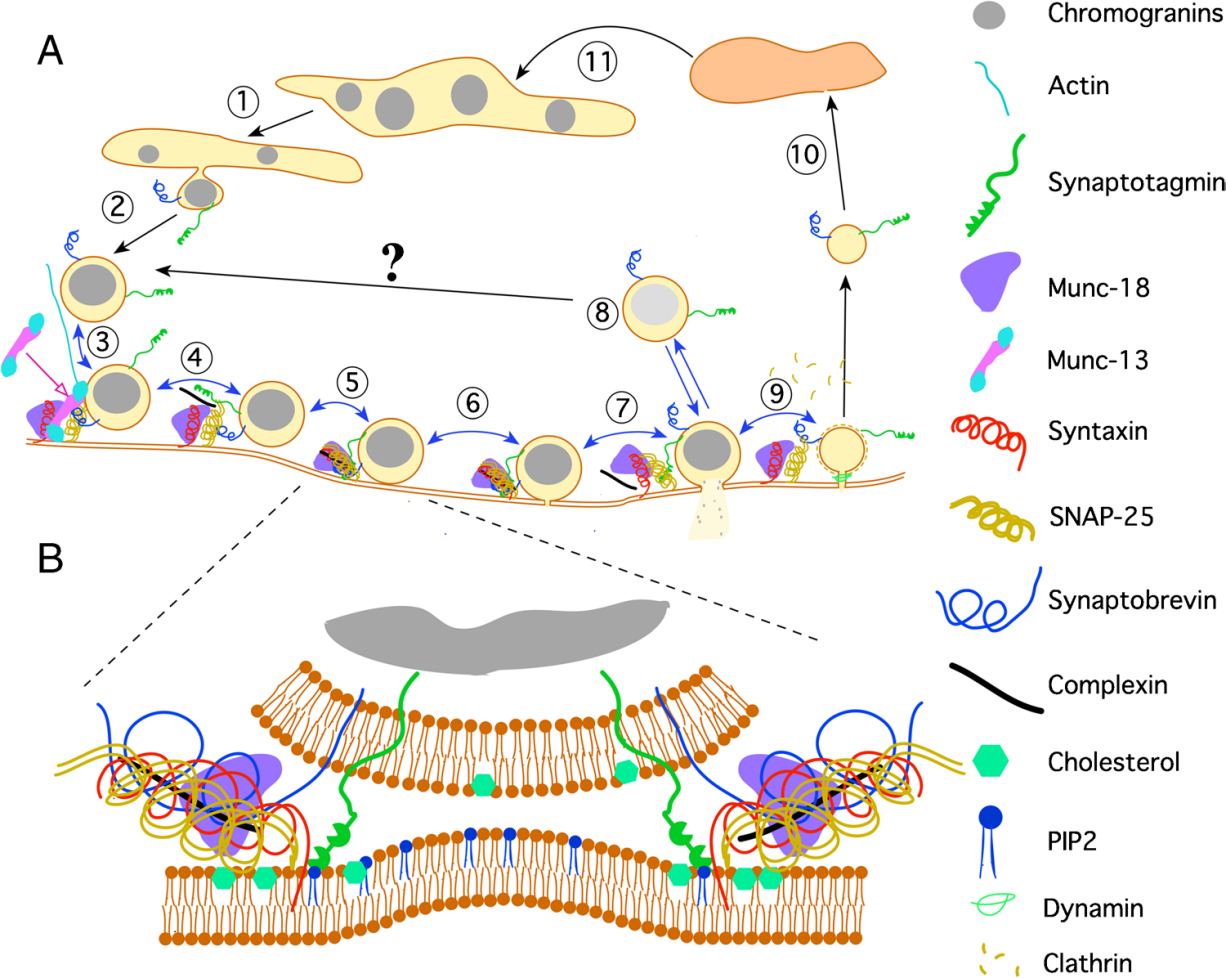
Exocytosis and endocytosis of CGs. **A**. Only one SNARE complex per granule is shown while some of the accessory proteins are omitted. (1) Granule proteins are sorted and packaged into the Golgi apparatus. (2) Granules are transported to the release sites by tubulin (not shown). (3) Actin filaments drive granules to specific tethering points and Munc-13 binds the complex and “opens” syntaxin. (4) “granule docking,” the SNARE complex starts to organize and the granule docks to the plasmalemma, Munc-13 is disassembled. Munc13 acts on syntaxin changing its conformation thereby leading to SNARE proteins zippering and the formation of fusion pore. (5) Priming, mediated by Munc-18 and complexin, thus allowing a tighter interaction of SNARE complexes. (6) The initial fusion pore allows a limited exchange of water and solutes. (7) Fusion pore dilatation allowing partial or complete release of the granule. The SNARE complex is disassembled. (8) Cavicapture. This partial exocytosis occurs when a dilated, but reversible, fusion pore allows the partial release of small molecules like peptides. Whether these granules can go to the SRP or get exocytosis again is unknown. (9) Membrane recovery by endocytosis. Two major mechanisms might be involved: depending on clathrin and dynamin 2, or by a clathrin-independent that uses dynamin 1 to promote granule fission. The inner content of the granule rapidly acidifies. (10) Clathrin is disassembled, and the granule can travel either deeper inside the cell towards either endosomes or lysosomes from where can re-enter in the secretory cycle after sorting in the Golgi. (11) Note that sizes of the resulted endocytotic granules are now smaller, and granule matrices are clearer as a result of protein loss during exocytosis. Note also that some of the steps are reversible (blue double head arrows). **B** Organization of the SNARE complex in the priming state. The presence of cholesterol and lysophospholipids allows the curvature of cell membrane. Note the lateral disposition of coiled-coil of proteins and the proposed situation of Munc-18 and complexin. Modified from [28]

It is generally accepted that SNARE proteins mediate membrane fusion by directed assembly of complementary SNARE motifs between the membranes destined to fuse (SNARE zippering). In his lecture at Hamburg, Reinhard Jahn discussed the possible scenarios for the structure and stabilization of the docked and primed states and the subsequent transition towards non-bilayer intermediates along the fusion pathway. Thus, membrane fusion occurs through distinct steps including docking, merger of proximal leaflets (stalk formation), and fusion pore formation. The structure of these intermediates is difficult to study because of their short lifetime. However, using the breakthrough interferometric scattering (iSCAT) technique, Jahn and coworkers observed that the diffusion coefficients of arrested vesicles decreased during progression through the intermediate states. Modeling allowed for predicting the number of tethering SNARE complexes upon loose docking and the size of the interacting membrane patches upon tight docking. These loosely and tightly docked states shed light on the nature of membrane-membrane interactions immediately before fusion [195]. A simplistic view of the different steps of membrane fusion and exocytosis is shown in Fig. 5.

Recent structural and single-molecule studies suggest the following pathway: syntaxin-1 and VAMP2 first bind on the surface of Munc18-1 to form a template complex, with which SNAP-25 associates to conclude SNARE assembly; these results demonstrate that stepwise SNARE assembly drives stagewise membrane fusion, Yongli Zhang concluded in his presentation [199]. An alternative view on SNARE assembly derives from a study of vesicle docking and mechanical interactions between SNARE proteins revealed by atomic force spectroscopy combined with fluorescence imaging. Vladimir Parpura presented data supporting the notion that native vesicle docking can be mediated by a single trans binary syntaxin1A-synaptobrevin2 complex without SNAP25 [113].

Josep Rizo presented a view on the underlying molecular mechanisms that exquisitely control neurotransmitter release. The accepted model involves the formation of SNAREs motifs of syntaxin-1, SNAP-25, and synaptobrevin, that are critical for exocytosis. Upon assembly, synaptotagmin-1 and complexin-1 bind to the SNARE complex, forming a primed state ready to induce fast membrane fusion upon Ca^2+^ binding to synaptotagmin-1. Using cryo-electron microscopy, the laboratory of Rizo has elucidated two structures of Munc18-1 bound to the SNARE complex. With molecular dynamics simulation, it was found that SNARES alone tend to induce the formation of these interfaces that together with the SNARE complex, form a spring-loaded macromolecular assembly that hinders premature fusion but is ready for fast fusion upon Ca^2+^ binding to synaptotagmin-1. Rizo presented a beautiful simulation of SNARE complexes as shown in recent articles [154, 155, 174].

Jacob Sørensen presented data suggesting that synaptotagmin-1 and synaptotagmin-7 when present alone, act as standalone fast and slow Ca^2+^ sensors for vesicle fusion in mouse CCs. When present together, they are found in largely non-overlapping clusters in LDCVs. These and other experiments suggest that synaptotagmin-7-dependent vesicle movement towards the membrane is involved in Munc13-2/phorbol ester/Ca^2+^-dependent priming as a prelude to fast and slow exocytosis triggering [2, 183].

Grant F. Kusick delivered a clear-cut didactic talk on how synaptotagmin-7 and Doc-2α influence release: do they enhance fusion of vesicles that are already docked and ready to fuse, or do vesicle dynamics play a role? His lab approached this question by monitoring of glutamate release and activity-dependent ultrastructural changes in neurons lacking each of these proteins. At small hippocampal synapses, Kusick proposed that synchronous release and asynchronous release after a single action potential result both from the fusion of docked vesicles; from a second action potential and onwards, fast vesicle docking regulated by synaptotagmin-7 becomes rate-limiting for all fusion and is therefore essential for both asynchronous release and facilitation [196]. Finally, the presentation of Ira Milosevic concerned amysin, a vertebrate-specific small SNARE protein that is engaged by phosphatidylinositol-4,5-bisphosphate to negatively regulate exocytosis.

By now, it seems clear that SNARE proteins are the major protagonists-driving membrane fusion; on the other hand, SNARE regulators provide the precision and temporal speed essential for Ca^2+^-triggered exocytosis. Also, intermediate steps of membrane fusion (priming, fusion triggering, and slow/rapid pore expansion) are being revealed from experiments in CCs, involving guiding of the vesicles from priming to fusion, pore opening and its expansion, and ensuing rapid cargo discharge with a sub-millisecond precision [45]. How many intermediate steps can be yet resolved in this sub-millisecond time range? Will the super-resolution techniques presented at Hamburg find new intermediates in the last stages of exocytosis?

### The fusion pore at a molecular level

At Hamburg, several communications dealt with the dynamics of the fusion pore at a molecular level. This gives an idea of the enormous progress of our knowledge of the exocytotic machinery and the fusion pore, ever since the lab of Eduardo de Robertis first reported an EM Ω-shaped picture of exocytosis in a CC [156]. This was obviously associated with super-resolution image techniques, as described in a session with experts from different companies. Thus, Gert Rapp presented the development of scanner-based laser systems in experiments using FRAP to understand intracellular transport processes. He also presented activities with 2- and 3-photon microscopy with 2-photon SLM-based photo-stimulation and FLUCS, a new technique for micro flow-induced photo-manipulation in living cells. Furthermore, Martin Oberhofer made a live presentation of the new Patch master software showing some features of capacitance measurements. Also, Steven Bump presented the orbital ring TIRF scanner that enables super-resolution applications such as single-molecule tracking.

The authors of this historical review were impressed by real-time images of single and compound fusion taken from live bovine CCs, using super-resolution stimulated emission depletion (STED) imaging of membrane dynamics, fusion pore dynamics, and vesicular content release dynamics, simultaneously in ~ 1500 living CCs. This study, presented by Ling-Gang Wu, established the sequence of sequential compound fusion with incredible super resolution images of single fusion pores, combined with Ca^2+^ currents and capacitance monitoring in single CCs. Wu concluded that sequential compound fusion and compound kiss-and-run (a new form of endocytosis) contribute to vesicle docking/priming, multivesicular release, asynchronous release, and endocytosis [65].

The different modes of exocytosis amperometrically analyzed revealed the variable nature of the fusion pore, as discussed by Meyer Jackson. Initially, the fusion pore is a rigid structure that may or may not expand to a flexible lipidic structure. If the pore does not expand, its closure occurs in a kiss-and-run process with a poor content release. Because the rate of fusion pore expansion depends on vesicle size, the choice between expansion and closure will also rely on vesicle size of an amperometric event, and the speed and composition of secretion [33].

Fluctuations of the fusion pore had been previously characterized with the elegant technique of patch amperometry in the laboratory of Manfred Lindau. The relation between amperometric detection of catecholamine release and membrane capacitance changes revealed that the amperometric foot signal preceding the amperometric spike reflected release through a narrow fusion pore with fluctuations of fusion pore conductance [4]. At Hamburg, Lindau presented a surprising finding obtained from experiments combining the amperometric recordings of individual exocytotic events using 4-electrode microfabricated electrochemical detector arrays and detection of such events with fluorescence imaging using TIRF microscopy. As expected, some amperometric spikes were preceded by the pre-spike foot signal and fluorescence event. However, surprisingly, in many other cases, apparent fluctuations of a foot signal preceding a spike were the result of overlapping catecholamine release activity at different locations, suggesting that distant exocytotic events and foot signals may occur in a synchronized manner. Thus, the interpretation of amperometric foot signals may seem less straightforward than typically assumed [43].

Another study approached the post-fusion release, and the capture of a vesicle membrane protein, the vesicular acetylcholine transporter (VAChT), was imaged from single vesicles in living PC12 cells, using super-resolution interferometric photo-activation localization microscopy and electron microscopy. Thus, the map to nanometer-scale topography and architecture of structures responsible for the transporter’s capture during exocytosis, could be modeled. When vesicles fuse with the plasma membrane, VAChT diffuses into the plasma membrane within a second, supporting a full-fusion exocytosis model. A portion of this material was then trapped over a dense matrix of clathrin-containing endocytic structures, ensuring that most of the transporter did not diffuse in the plasma membrane more than several hundred nanometers. Modeling supports the idea that a simple system of randomly distributed traps at high density can account for the limited spread of vesicle material and its recapture [145, 172]. In line with this previous study, Justin Taraska presented a correlative super-resolution technique that combined light and EM methods to produce images where identified proteins are mapped with the dense native environment of the plasma membrane and associated organelles at the molecular scale. Using these techniques, it was observed that Rab-GTPases and their effectors are distributed across the entire surface of individual docked vesicles. Taraska suggested that this circumferential distribution likely aids in the efficient transport, capture, docking, and rapid fusion of vesicles in excitable cells. A recent study co-authored by Taraska, Uri Asheri, and Wu’s groups concluded that clathrin-mediated endocytosis collaborates with bulk endocytosis to augment endocytic capacity in active secretory cells [7].

### Altered functions of chromaffin cells and SNARE proteins in disease

Since the ISCCB-1 meeting of Ibiza in 1982, an increasing number of functions have been discovered for proteins and nanopeptides stored in the LCDVs of neuroendocrine cells. Thus, the laboratory of Karen Helle first observed that vasostatin (the N-terminal domain of chromogranin A) diminished the contraction in isolated segments of human blood vessels [1]. A few years later, Dominique Aunis and coworkers isolated secretolytin, a peptide of the C-terminal sequence of chromomembrin that exhibited antibacterial activity [176]. Since then, several new peptides have been isolated, and some of their regulatory properties on various organs have been identified.

At Hamburg, Sushil K. Mahata discussed catestatin and its mimetics as potential therapies for hypertension, diabetes, and gut motility disorders. Curiously, Sahar El Aidy also discussed the regulation by catestatin of gut bacterial taxa with selective antimicrobial resistance. Additionally, Carmine Rocca commented on chromogranin A as a cardioregulatory hormone acting on neuropilin-1 receptor, as well as on its ability to exert cardioprotection against doxorubicin-dependent cardiotoxicity [157].

In spontaneously hypertensive rats (SHRs), CCs secrete higher amounts of catecholamines in response to depolarizing stimuli, as independently revealed by experiments from the labs of Antonio G. García [124] and Arturo Hernández-Cruz [167]. In Hamburg, a collaborator of Arturo, Óscar J. Parada-Parra, presented new experiments concerning the ability of the endoplasmic reticulum (ER) to behave as a “Ca^2+^ source” and the higher secretion of catecholamines in SHR rats, compared to respect to normotensive Wistar-Kyoto rats. Altered secretion has also been reported in diabetic rats [134] and during hypoxia that, curiously, augments the expression of Cav3.2 T-type channels that trigger “low-threshold” exocytosis in adult rat CCs [27].

Low oxygen pressure is an environmental stressor that triggers catecholamine release from CCs [91]. CCs in the fetus and the newborn are particularly sensitive to hypoxia; in this condition, catecholamine release is critical for survival [168]. Such release is triggered by hypoxia-induced blockade of K^+^ channels, leading to membrane depolarization, cell firing, and Ca^2+^ entry through Cav channels [125]. The mechanisms underlying hypoxia-elicited secretion involves complexes I and IV of the mitochondria electron transport chain, coupled to the alteration of reactive oxygen species [126]. Other numerous contributions, towards the classification of the ion channels and signaling pathways on catecholamine release during acute or chronic hypoxia, are described in the reviews mentioned above.

Interest in exploring the potential functional alterations of adrenal CCs in neurodegenerative diseases has been steadily increasing during the last decade. This has been investigated following two strategies namely, the effects on exocytosis of pathological proteins typically associated to such conditions and expressed in CCs or through the study of CC functions in transgenic mouse models of neurodegenerative diseases.

Aggregates of α-synuclein are found in familiar Parkinson’s disease (PD). The overexpression of this protein in mouse CCs decreases secretion by acting at a late step of exocytosis [104] associated with an acceleration of exocytosis due to fusion pore dilation [115]. Also, in patients over-expressing α-synuclein altered functions in their adrenal medulla and CCs, that also express the protein, have been discovered [62].

At Hamburg, an original question linked to a peripheral blood marker for PD was raised by Ricardo Borges. As blood platelets use mechanisms to accumulate serotonin in secretory vesicles similar to those used by striatal neurons to accumulate dopamine, the lab of Borges investigated whether blood platelets from PD patients had an altered mechanism for serotonin transport. He concluded that PD patients have a functional impairment of the serotonin handling by platelets that could serve as a biomarker for disease diagnosis at presymptomatic stages [127]. Another communication by Uri Asheri dealt with the detection of α-synuclein aggregates in skin biopsies of PD patients, using super-resolution microscopy; this could serve as biomarker to explore early changes in the prodromal stage of PD. Still, on the same topic, Mau Sharma commented on the release of pathogenic α-synuclein through SNARE-dependent lysosomal exocytosis, proposing a central mechanism for exocytosis of aggregated and degradation-resistant proteins from neurons, that could contribute to disease propagation. One more communication by Valentina Carabelli dealt with the recording of the activity of midbrain dopamine neurons using micro-graphitic diamond multi-electrode arrays (D-MEAs). With a new specially designed dual-mode electronic front-end, it was possible to monitor the simultaneous detection of exocytotic events and action potential from cultured neurons. α-synuclein oligomers induced a progressive impairment of spontaneous firing and reduced in the network synchronization, Valentina pointed out.

Concerning Huntington’s disease (HD), the huntingtin-associated protein 1 (HAP1) that CCs endogenously express reduces full fusion exocytosis by affecting vesicle docking and controlling fusion pore stabilization [116]. Additionally, HAP1 also decreases endocytosis by interacting with some proteins of the exocytosis machinery and its binding to clathrin [117]. Furthermore, in CCs of the R6/1 [122] and R6/2 [88] mice models of HD, a pronounced decrease of quantal catecholamine release with other alterations in ion currents and cell excitability have been reported. This was also the case for the APP/PS1 mouse model of Alzheimer’s disease (AD) [49]. The SOD1^G93A^ mouse model of amyotrophic lateral sclerosis (ALS) has also been explored. In Hamburg, ultrastructural alterations and function of mitochondria (Antonio García) and CGs (Fernando Padín) in CCs from SOD1^G93A^ mice were reported [123]. Of note is a report on altered mitochondrial metabolism and reduced secretion in CCs from the TS2-neo mouse model of autism [24].

Some curious examples of translational medicine concerning a secretory process were presented in Hamburg. One concerned the exocytotic secretion of surfactant from lamellar bodies of alveolar epithelial cells, mediated by a fusion pore expansion linked to Ca^2+^ binding to synaptotgmin-7, a mechanism likely disturbed in pathological situations of alveolar edema, Manfred Frick commented. An excellent basic-clinical work related to the prevention of mucin hyper-secretion with drug therapy potential in asthma, chronic obstructive pulmonary disease, respiratory viral infections, and cystic fibrosis was presented by Axel T. Brunger. In a reconstituted system with proteins of the airway secretion machinery (syntaxin-3, SNAP23, VAMP8, synaptotagmin-2, along withMunc13-2 and Munc 18–2), a synthetic hydrocarbon-stapled peptide interfering with synaptotagmin, strongly suppressed Ca^2+^-triggered fusion and attenuated mucus occlusion in mouse airway [103]. Human pathogenic mutations of SNAREs (SNAREopathies) and their key regulators give rise to various neurodevelopmental disorders, suggesting that such proteins may be drugable to develop therapies for those diseases [188]. In this context, Jacqueline Burré commented on missense mutations of Munc18-1 or reduced levels of this protein, that are linked to epilepsies, intellectual disability, movement disorders, and neurodegeneration [70][70]. Furthermore, Geert van der Bogaart presented a mutation in the STX5 gene (coding for syntaxin-5) that gives rise to severe pathologies including abnormal glycosylation, skeletal disorders, and very short survival. In fibroblasts from these patients, he said that the short form of syntaxin-5 is the dominant SNARE for intra-Golgi transport [111]. Another communication linked to lysosomes storage diseases, reported data on the accumulation of cholesterol in secretory vesicles with concomitant smaller fusion pore conductance, as Jernej Jorgacevski concluded.

**Fig. 6:**
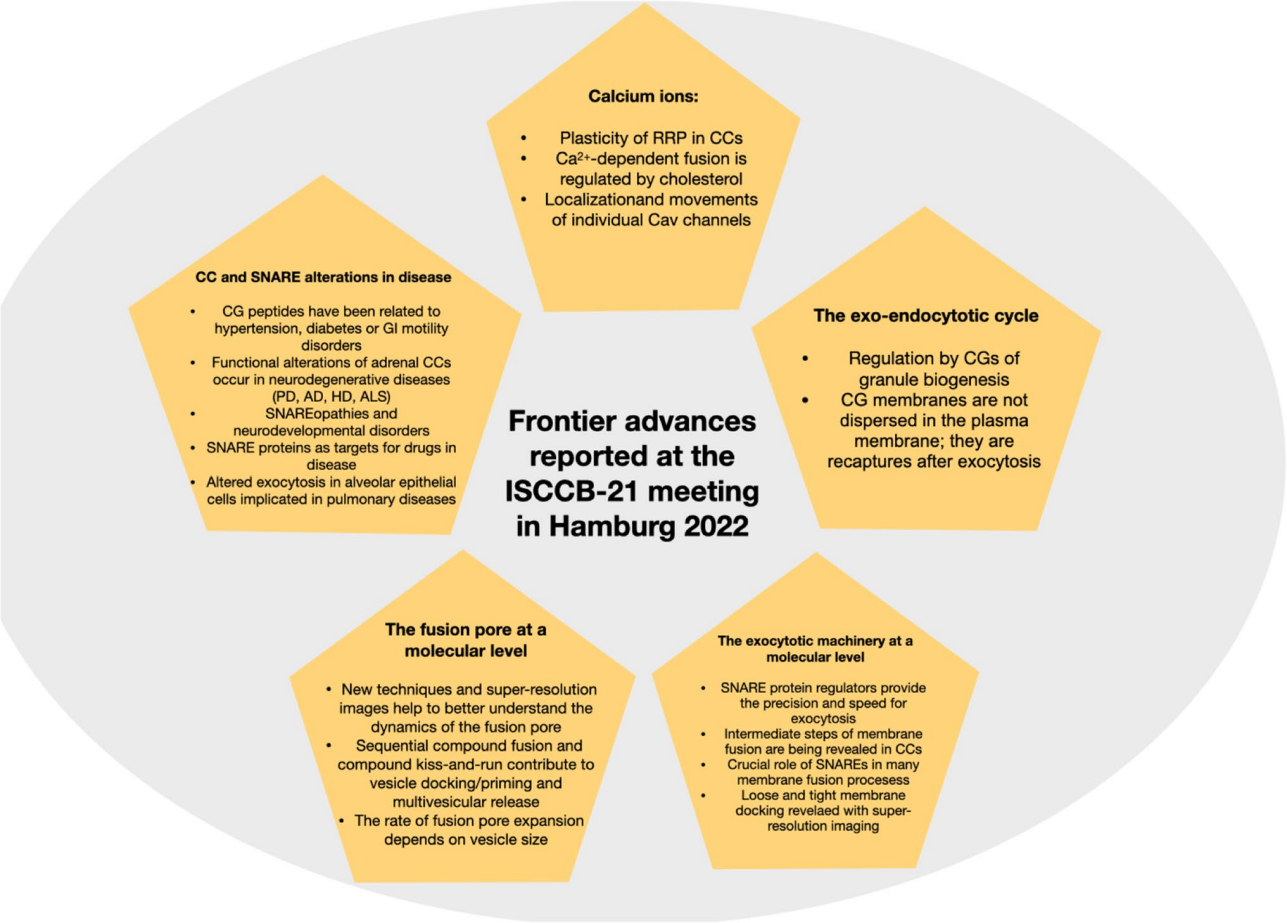
Some frontier advances reported at the ISCCB-21 meeting in Hamburg. CC, chromaffin cell; CG, chromaffin granule; RRP, readily release vesicle pool; SNARE, soluble NSF attachment protein receptor

Some of the frontier advances reported in the ISCCB-21 meeting are summarized in Fig. 6.

## Conclusions and perspectives

Here we have presented a historical view on the evolution of knowledge on the structure and function of the mammalian adrenal medulla and its CCs. Two time periods have been analyzed: the first one extends from around the middle of the nineteenth century to 1982, the year when the first ISCCB meeting was held at the Spanish island of Ibiza; and the second comprises the period from 1982 to 2022 when the 21st ISCCB meeting was held at Hamburg, Germany. The first period witnessed the clarification of the basic mechanisms of catecholamine synthesis, their storage in CGs, the exocytotic Ca^2+^-dependent release of adrenaline and noradrenaline during stressful conflicts, and the sympathetic-like nature of CCs. The second period, heralded by the 21 biennial ISCCB meetings held around the world during the last 40 years, witnessed extraordinary advances in cell excitability, the identification and characterization of the multiple ion channels expressed by these cells including all the Cav channels described in neurons, the dynamics of Ca^2+^ entry and its redistribution into intracellular organelles, the characterization of the SNARE proteins and their regulatory proteins in the processes of exocytosis, the formation of the fusion pore and its expansion, and the various alterations of CC functions in cardiovascular, metabolic, and neurodegenerative diseases.

In addition to their intrinsic physiological and pathological interest, it has been notable the use of CCs as a neuronal model to explore the basic mechanisms underlying the exocytotic machinery, the fusion of membranes, the formation of the fusion pore, and its expansion (exocytosis) and retraction (endocytosis). This was nicely illustrated in the program that Manfred Lindau built up on the occasion of the 21st ISCCB meeting in Hamburg, last August 2022. Of high interest were the presentations linked to various cell types on the diversity of protein subtypes of the SNARE complexes, related to superfast exocytosis (synapses), fast exocytosis (chromaffin cells), slow exocytosis (beta cell), or very slow exocytosis (mucin secretion). This universal secretory machinery is giving rise, on the one hand, to the identification of mutations linked to disease and on the other, to the identification of targets with potential to develop new ligands with therapeutic purposes.

An increasing number of physiological effects are being recognized in the proteins co-stored with catecholamines in CGs, particularly of peptides derived from chromogranins. The interest in this topic is growing as the proteic cargo of CGs is relevant. This is the case of opiates that are co-stored and released into the circulation with adrenaline; in so doing, opiates may have an analgesic function during stressful conflicts.

**Fig. 7:**
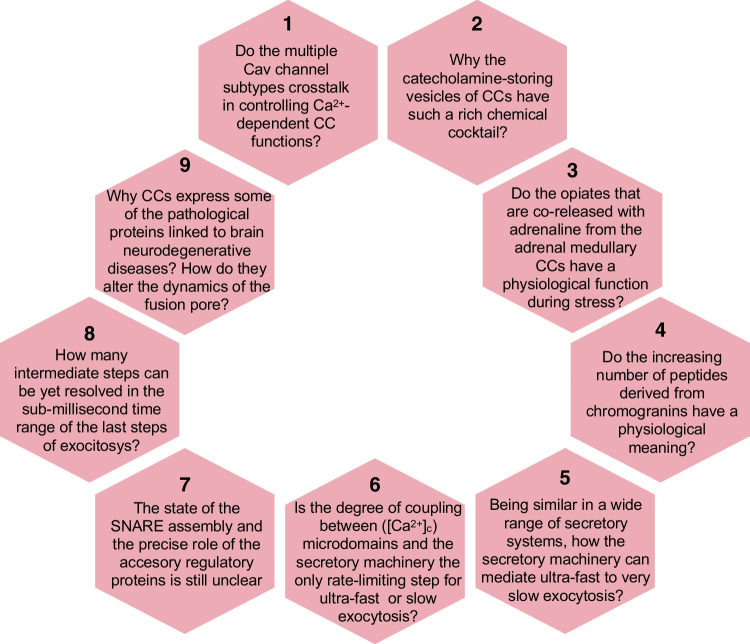
Some unresolved question that emanated from the ISSCB-21 meeting at Hamburg concerning CC biology

We predict that CCs, readily cultured from mammals of any age, will continue to be central in exploring basic problems on cell excitability, ion channels, Ca^2+^ dynamics, membrane fusion, and exocytosis in the following years. Some relevant unresolved questions concerning these topics are enumerated in Fig. 7. Surely, we will see some answers to them at the 22nd ISCCB meeting in Israel, organized by Uri Ashery, in 2024.

## Data Availability

Not applicable.

## Abbreviations

CC: Chromaffin cell
CG: Chromaffin granule
Cg: Chromogranin
ISCCB: International Symposium on Chromaffin Cell Biology
LDCV: Large dense-cored vesicles
SNARE: Soluble NSF attachment protein receptor
vSNARE: Vesicle SNARE
